# Effects of environmental feedback on species with finite population

**DOI:** 10.1016/j.isci.2024.109055

**Published:** 2024-01-26

**Authors:** Jia-Xu Han, Rui-Wu Wang

**Affiliations:** 1 School of Ecology and Environment, Northwestern Polytechnical University, Xi’an 710072, P.R. China; 2 Zoology Department and Biodiversity Research Centre, University of British Columbia, Vancouver, British Columbia V6T 1Z4, Canada

**Keywords:** Natural sciences, Biological sciences, Evolutionary biology

## Abstract

In an unchanging environment, natural selection always selects species with high fitness. In this study, we build a co-evolutionary system to study the interaction between stochasticity in finite populations and environmental feedback. Positive feedback between species and environment is detrimental to the invasion success, whereas negative feedback is beneficial to invasion since feedback makes population size important enough to revise natural selection’s preference. In competition scenario, positive and negative feedback will benefit the initially inferior species. When selection intensity is high, negative feedback may even cause natural selection to favor the initially inferior species. All of these effects are caused by feedback that allows the initially inferior species to have greater fitness than the initially dominant species. Our results emphasize that the effects of stochasticity in evolutionary path can be reinforced by feedback with environment and then reverse the preference of natural selection.

## Highlights

• Early random accidents are important due to feedback between environment and species
• Positive feedback makes invasion difficult and negative feedback makes invasion easy
• Negative feedback can revise the favor of natural selection in competition scenario

## Graphical abstract

**Figure:**
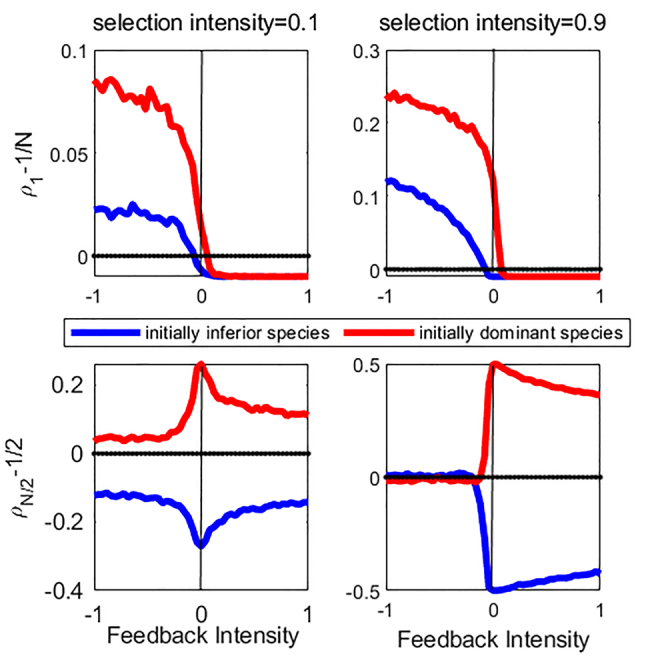

## Introduction

Natural selection of Darwin implies that biological organisms are evolved gradually in a direction to increase fitness due to selfness of organisms, which is an idea of rationality of human being proposed by Adam Smith.^1^^,^^2^ In this theoretical framework, the individuals or the populations will reach to equilibrium and therefore predictable through the competition among each other, especially when the shared resource is saturated, and it is also the fundamental concept of Modern Synthesis in theory of evolutionary biology.^3^^,^^4^ Competition as the mechanism for the nature selection pushes evolution toward optimum fitness, which also constitutes a balance. Ecology and evolution research nowadays frequently highlights this balanced perspective of dynamic systems by resolving a model that identifies and defines the stability of their balances.^5^ The mathematical ecologists generally ignore history accidents since they believed it was smacked of particularities and had little generality.^6^

However, the historical accidents might play an important role in evolutionary process that even might change the species speciation or population and ecosystem structure such as priority effect. The first species to arrive in a habitat alters the resources available to other species, making the environment more or less appropriate for them.^7^^,^^8^ Historical accidents described by the evolutionary path often simultaneously experience a feedback process of adaptation in which the organisms can both change and be affected by their environment, and are both subjects and producers of natural selection.^9^^,^^10^^,^^11^ For example, the organisms can change the environment by their metabolism, activities and choices.^12^^,^^13^ A change at any point in the sequence of historical accidents can change the environment thereafter, and thus affect the natural selection that organisms face afterward. These adaptation feedback in environment may function as negative feedback, which usually lead to equilibrium and predictable results, or positive feedback, which allows for evolution to adapt faster than evolution by selection from the unchanging environment.^14^^,^^15^ The positive feedback causes every stage of development depends on its preceding phases.^16^ Then, any historical event may affect the subsequent evolution process and the sequence of history has a major impact on the result.^17^^,^^18^ In contrast, negative feedback is commonly associated with equilibrium state, where any deviation from the equilibrium is anticipated to return to the equilibrium through negative feedback.^19^

In evolutionary biology, stochasticity in the evolutionary process is integral part of population genetic theory and phylogenetic modeling when the population size is finite. One phenotype is frequently discovered to be marginally beneficial over another phenotype and one of these phenotypes may be selected according to stochastic process.^20^ Even traits that are not favored by natural selection have the ability to take over a whole population, which is a key distinction between deterministic and stochastic models of evolution.^21^ It is worth noting that in finite population dynamics, individual births and deaths are stochastic. As a result, in finite population dynamics, any change at any previous moment can be viewed as a historical accident.

Weitz et al.^22^ reveals a fascinating cyclical tragedy dynamic in which the system cycles between depletion and replete environmental states and cooperation and defection behavior states by considering environment dependent payoffs and strategies coevolve. After that, there has been a growing body of research on the role of feedback with environment in the evolutionary process to involve punishment, imitation strategy, and aspiration dynamic in Weitz’s framework.^23^^,^^24^ However, there has been little research into the impact of environmental feedback on a finite population. To emphasize the significance of the interplay of feedback with environment and historical accidents, we employ a finite population model to explore the influence of feedback with the environment on the probability of a species taking over the whole population. To begin, we construct a co-evolutionary process between species and environment. Then, in the absence of feedback, we compute the analytic solution for the finite population dynamics. Finally, we incorporate feedback and demonstrate how it works using individual-based simulation.

## Model

In this section, we look at how species and their environments co-evolve. The term “species” in this sense includes not only species abundance but also gene frequency or other relevant features. It might also be a trait or a mutation. The term “environment” might refer to a resource that benefits the species, and we will use “resource” instead of “environment” later in the text to simplify the explanation. Whether the resource is created by the species or just used by the species, there will be positive or negative feedback between the species and the environment. We assume that the two resources are similar in nature, such as two chemicals, bacteria or plants, thus making their quantities directly comparable. To keep things simple, we disregard the influence of density dependence on species and resource dynamics. As an example, consider two species with two resources.

First, we explore the role of the resource in the co-evolutionary process. We assume that there are no relationships between the resources, allowing us to study their dynamics individually. This might be a microcosm of each species’ own resource, while they share certain resources. We propose that the dynamic of the resource itself obeys exponential growth, similar to the generalized Lotka-Volterra equation, which is a frequently used tool for understanding the dynamics of interacting populations.^25^ The more contacts there are or the more resources there are, the faster the resources and species are eaten, and the more suitable it is for that resource to increase. The resource dynamic may thus be expressed as:

$$\left\{ \begin{array}{c} \frac{dY_{1}}{dt}=Y_{1}\left(a_{1}X_{1}+b_{1}\right)\\ \frac{dY_{2}}{dt}=Y_{2}\left(a_{2}X_{2}+b_{2}\right)\text{,} \end{array}\right.$$

where $Y_{i}$ represents the amount of resource *i*, $X_{i}$ represents the number of individuals of the species *i* that is paired with resource *i*, $a_{i}$ represents the influence of species presence on resource growth rate, and $b_{i}$ represents per unit resource growth rate with the loss of species *i*. To indicate the ratio of total resources for resource *i*, we may use $y_{i}=\frac{Y_{i}}{Y_{1}+Y_{2}}$. The dynamic of $y_{i}$ then follows:

$$\left(Y_{1}+Y_{2}\right)\frac{dy_{i}}{dt}=\frac{d\left(Y_{1}+Y_{2}\right)y_{i}}{dt}-\frac{d\left(Y_{1}+Y_{2}\right)}{dt}y_{i}=\frac{dY_{i}}{dt}-\frac{d\left(Y_{1}+Y_{2}\right)}{dt}y_{i}=Y_{i}\left(a_{i}X_{i}+b_{i}\right)-y_{i}\sum_{j=1}^{2}Y_{j}\left(a_{j}X_{j}+b_{j}\right).\;i=1,2\text{.}$$

Then, we can rewrite the dynamic of $y_{i}$ as

(Equation 1) $$\left\{ \begin{array}{c} \frac{dy_{1}}{dt}=y_{1}\left(1-y_{1}\right)\left(a_{1}X_{1}+b_{1}-a_{2}X_{2}-b_{2}\right)\\ \frac{dy_{2}}{dt}=y_{2}\left(1-y_{2}\right)\left(a_{2}X_{2}+b_{2}-a_{1}X_{1}-b_{1}\right)\text{.} \end{array}\right.$$

Because we are interested in the effect of feedback, we simplify the model by assuming that the values of the parameters for these resources are equivalent, i.e., $a_{1}=a_{2}=a$ and $b_{1}=b_{2}$. We can also get

Equation 2 $$\left\{ \begin{array}{c} \frac{dy_{1}}{dt}={ay}_{1}\left(1-y_{1}\right)\left(X_{1}-X_{2}\right)\\ \frac{dy_{2}}{dt}={ay}_{2}\left(1-y_{2}\right)\left(X_{2}-X_{1}\right) \end{array}\right.$$

The parameter *a* can be used to quantify the qualities of feedback between environment and species, with a>0 indicating positive feedback, a=0 indicating no feedback, and a<0 indicating negative feedback. And the |a| can quantify the intensity of feedback between the environment and the species.

We assume that the overall population size is fixed and finite in each step of the process, and that only one new member is produced and one member is chosen to die (birth-death process) in each time step, which is a general stochastic process based on Moran’s model in the presence of demographic stochasticity.^26^ The new member is assigned the same phenotype as one randomly selected existing member, with a probability equal to the proportion of that member’s fitness divided by the total fitness of all members. And the member chosen to die is picked at random, with a uniform distribution across the species. Because the union resource *i* will only be used by species *i*, the advantages received by species *i* are $f_{i}=c_{i}y_{i}+d_{i}$. Because the species gain from the shared resources, the union resource only makes a minor contribution to fitness, which is emphasized by weak selection. Weak selection describes situations in which reward differences have minimal influence, allowing random fluctuations to dominate evolutionary dynamics.^27^^,^^28^ The fitness of species *i* will be $F_{i}=1-w+wf_{i}$, where *w* indicates the intensity of selection.

In this context, we evaluate the probability $\rho_{k}$ of a species i=1 with *k* individuals taking over a population with a total population of *N* individuals. A similar scenario exists for the species i=2. Then we look at two scenarios. To begin, we evaluate the fixation probability $\rho_{1}$ of species 1, which is the probability that species 1 will ultimately take over the whole population when the population initially comprises just one individual from species 1 and all other individuals are from species 2. This condition is analogous to when a species accidentally invades another species’ environment or when a mutant develops by chance in a population (invasion scenario). The fixation probability will then be the probability that the invader or mutation will take over the whole population. The alternative conditions is $\rho_{\frac{N}{2}}$, which is the probability that species 1 will ultimately take over the whole population if the population begins with half of the individuals from species 1 and half of the populace from species 2. This circumstance relates to a fair competition between the two species, such as the dynamics at the intersection of the distribution zones of two species (competition scenario). In this fair fight, the probability $\rho_{\frac{N}{2}}$ denotes the probability that species 1 will defeat species 2.

## The situation without feedback

To begin, we investigate the case when there is no feedback between species and environment, which is a=0, as a baseline for future outcomes. The co-evolution system will then degenerate into a simple finite population process, and we will set $y_{1}=y_{2}=1/2$. In each phase of the Moran process, one individual is randomly picked to produce offspring and one individual is randomly selected to die. The possibility of species 1 with *k* individuals increasing to *k+1* individuals is the probability of an individual of species 1 chosen to birth multiplied by an individual of species 2 chosen to die, and it may be represented as

$$p_{k}^{+}=\frac{kF_{1}}{kF_{1}+\left(N-k\right)F_{2}}\frac{N-k}{N}\text{.}$$

The possibility of species 1 with *k* individuals drops to *k-1* individuals is the probability that an individual of species 2 chosen to be born times an individual of species 1 chosen to die, and it may be represented as

$$p_{k}^{-}=\frac{\left(N-k\right)F_{2}}{kF_{1}+\left(N-k\right)F_{2}}\frac{k}{N}\text{.}$$

Except for the absorbing states, $\rho_{0}=0$ and $\rho_{N}=1$, the dynamic of this finite population will follow

$$\rho_{k}=\rho_{k+1}p_{k}^{+}+\rho_{k}\left(1-p_{k}^{-}-p_{k}^{+}\right)+\rho_{k-1}p_{k}^{\_}\text{,}$$

because the condition of *k* individuals originate from three circumstances: *k+1* individuals reduce one individual, *k* individuals remain stable, and *k-1* individuals grow one individual. To solve this equation, we follow Nowak^21^ and Ohtsuki et al.^29^ Let $\alpha_{k}=\rho_{k}-\rho_{k-1}$ and the dynamic of this finite population be written as $\alpha_{k+1}=\frac{p_{k}^{-}}{p_{k}^{+}}\alpha_{k}$. Because $\sum_{k=1}^{N}\alpha_{k}=\rho_{N}-\rho_{1}=1$ and $\alpha_{k}=\prod_{j=1}^{k-1}\frac{p_{j}^{-}}{p_{j}^{+}}\rho_{1}$, we may have $\rho_{k}=\frac{1+\sum_{j=1}^{k-1}\prod_{l=1}^{j}\frac{p_{j}^{-}}{p_{j}^{+}}}{1+\sum_{j=1}^{N-1}\prod_{l=1}^{j}\frac{p_{j}^{-}}{p_{j}^{+}}}$.

Because the selection is weak (w≪1) in the invasion scenario, we may calculate an approximation of the fixation probability,

Equation 3 $$\rho_{1}=\frac{1}{1+\sum_{j=1}^{N-1}\prod_{l=1}^{j}\frac{p_{j}^{-}}{p_{j}^{+}}}=\frac{1}{1+\sum_{j=1}^{N-1}\prod_{l=1}^{j}\frac{F_{2}}{F_{1}}}=\frac{1}{1+\sum_{j=1}^{N-1}\prod_{l=1}^{j}\frac{1-w+wf_{2}}{1-w+wf_{1}}}=\frac{1}{1+\sum_{j=1}^{N-1}\prod_{l=1}^{j}\left(1+w\frac{f_{2}-f_{1}}{1-w+wf_{1}}\right)}\approx \frac{1}{1+\sum_{j=1}^{N-1}\prod_{l=1}^{j}\left[1+w\left(f_{2}-f_{1}\right)\right]}\approx \frac{1}{1+\sum_{j=1}^{N-1}\left[1+\sum_{l=1}^{j}w\left(f_{2}-f_{1}\right)\right]}=\frac{1}{N+w\frac{N\left(N-1\right)}{2}\left(f_{2}-f_{1}\right)}=\frac{1}{N}\frac{1}{1+w\frac{\left(N-1\right)}{2}\left(\frac{c_{2}}{2}+d_{2}-\frac{c_{1}}{2}-d_{1}\right)}\text{.}$$

If the process is completely random, which implies that all of the parameters of species 1 and species 2 are the same, the probability of species 1 originally containing k individuals taking over the population is $\frac{k}{N}$. Then, if $\rho_{1}>\frac{1}{N}$, we may argue that natural selection favors species 1, which is equal to

Equation 4 $$f_{2}=\frac{c_{2}}{2}+d_{2}<\frac{c_{1}}{2}+d_{1}=f_{1}\text{.}$$

**Figure 1:**
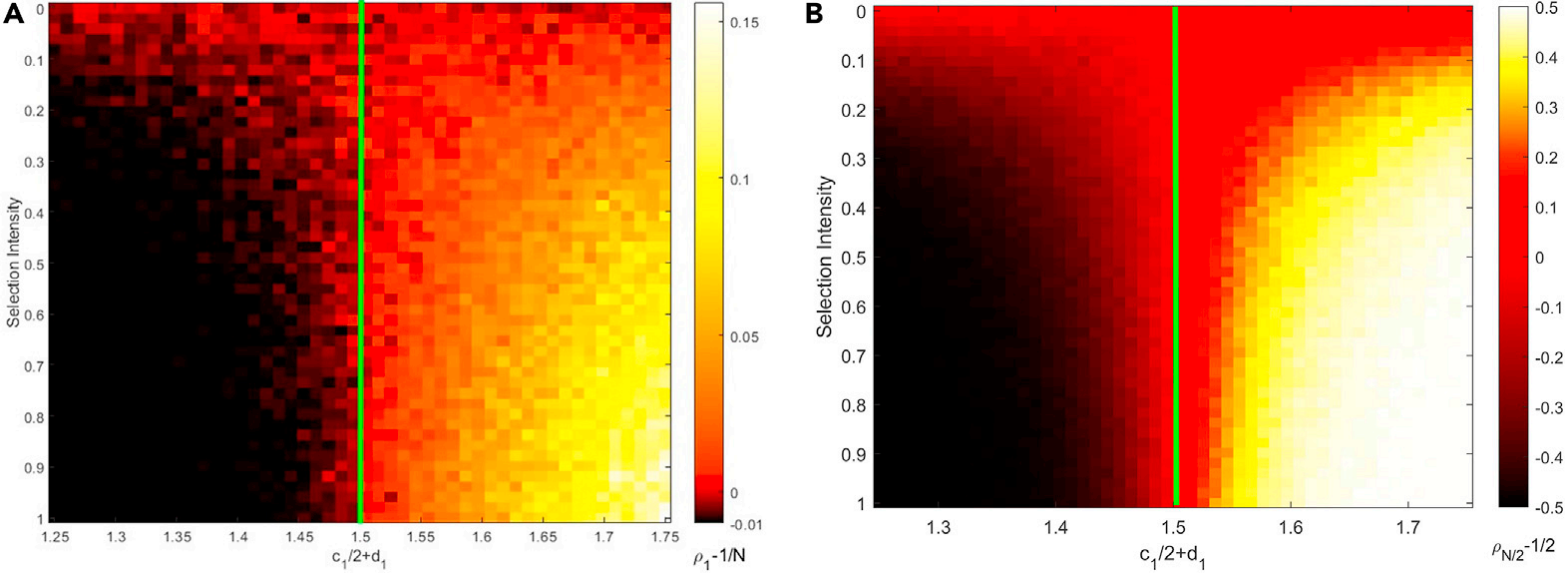
The degree of natural selection preference in the simple situation without feedback between environment and species (A and B) The green line indicates the case where $\frac{c_{1}}{2}+d_{1}=\frac{c_{2}}{2}+d_{2}$. For both (A) invasion scenario and (B) competition scenario, nature selection prefers species 1 when $\frac{c_{1}}{2}+d_{1}>\frac{c_{2}}{2}+d_{2}$ is satisfied and species 2 when $\frac{c_{2}}{2}+d_{2}>\frac{c_{1}}{2}+d_{1}$ is satisfied. The degree of natural selection preference increases with the selection intensity. The entire population size is 100. Each simulation is conducted a total of 10000 times to compute the probabilities $\rho_{\frac{1}{2}}$ and $\rho_{\frac{1}{N}}$, and data are represented as mean.

Because the environment remains constant, it is simple to grasp that natural selection favors species only if the fitness of species 1 is greater than that of species 2. In the competition scenario, we get $\rho_{\frac{N}{2}}\approx \frac{1}{2}\frac{1+w\frac{\left(N/2-1\right)}{2}\left(\frac{c_{2}}{2}+d_{2}-\frac{c_{1}}{2}-d_{1}\right)}{1+w\frac{\left(N-1\right)}{2}\left(\frac{c_{2}}{2}+d_{2}-\frac{c_{1}}{2}-d_{1}\right)}$. In this case, $\rho_{\frac{N}{2}}>1/2$, which is also identical to inequality (4), suggests that species 1 has a higher probability of dominating the population than species 2. In other words, natural selection only favors species with high fitness in the simple situation without feedback between environment and species. To replicate this basic Moran process, we also utilize an individual-based model to simulate the degree of natural selection preference, which is defined as $\rho_{1}-\frac{1}{N}$ for invasion scenario and $\rho_{\frac{N}{2}}-\frac{1}{2}$ for competition scenario (Figure 1). In the zone satisfying inequality (4), the values of $\rho_{1}-\frac{1}{N}$ and $\rho_{\frac{N}{2}}-\frac{1}{2}$ are positive, which is consistent with our prior analysis. In addition, the intensity of selection raises the degree of natural selection preference. This is because an increase in the selection intensity results in higher fitness and birth rate for the dominant species, which in result leads to a higher probability of taking over the entire population. Then, we refer to species 1 as the initially dominant species if $\frac{c_{1}}{2}+d_{1}>\frac{c_{2}}{2}+d_{2}$ is fulfilled, initially inferior species if $\frac{c_{2}}{2}+d_{2}>\frac{c_{1}}{2}+d_{1}$ is satisfied, and initially neutral species if $\frac{c_{2}}{2}+d_{2}=\frac{c_{1}}{2}+d_{1}$ is satisfied.

## The situation with feedback

When the presence of a species has an effect on the environment, which means a≠0, stochastic processes in finite populations then have an impact on the environment. Changes in the environment will then alter the fitness of the species and, as a result, the stochastic processes of the finite population. Because of the feedback, this co-evolution system has become so complicated that an analytical solution cannot be provided. And we will continue to analyze this system utilizing individual-based simulation. To simulate, we must rewrite the resource dynamic (Equation 2) as:

Equation 5 $$\Delta y_{i}={ay}_{i}\left(1-y_{i}\right)\left(X_{i}-X_{3-i}\right)\Delta t\text{,}$$

where Δt is the time step for modeling differential equations and $\Delta y_{i}$ represents the resource change per time step. Δt is also the timescale between the dynamics of species and environment in this case. And we set the beginning resources for both species to be averaged. The simulations on the species component are consistent with the preceding section, and we assume that the change of species occurs first, followed by the change of environment in one time step.

### The situation with positive feedback

**Figure 2:**
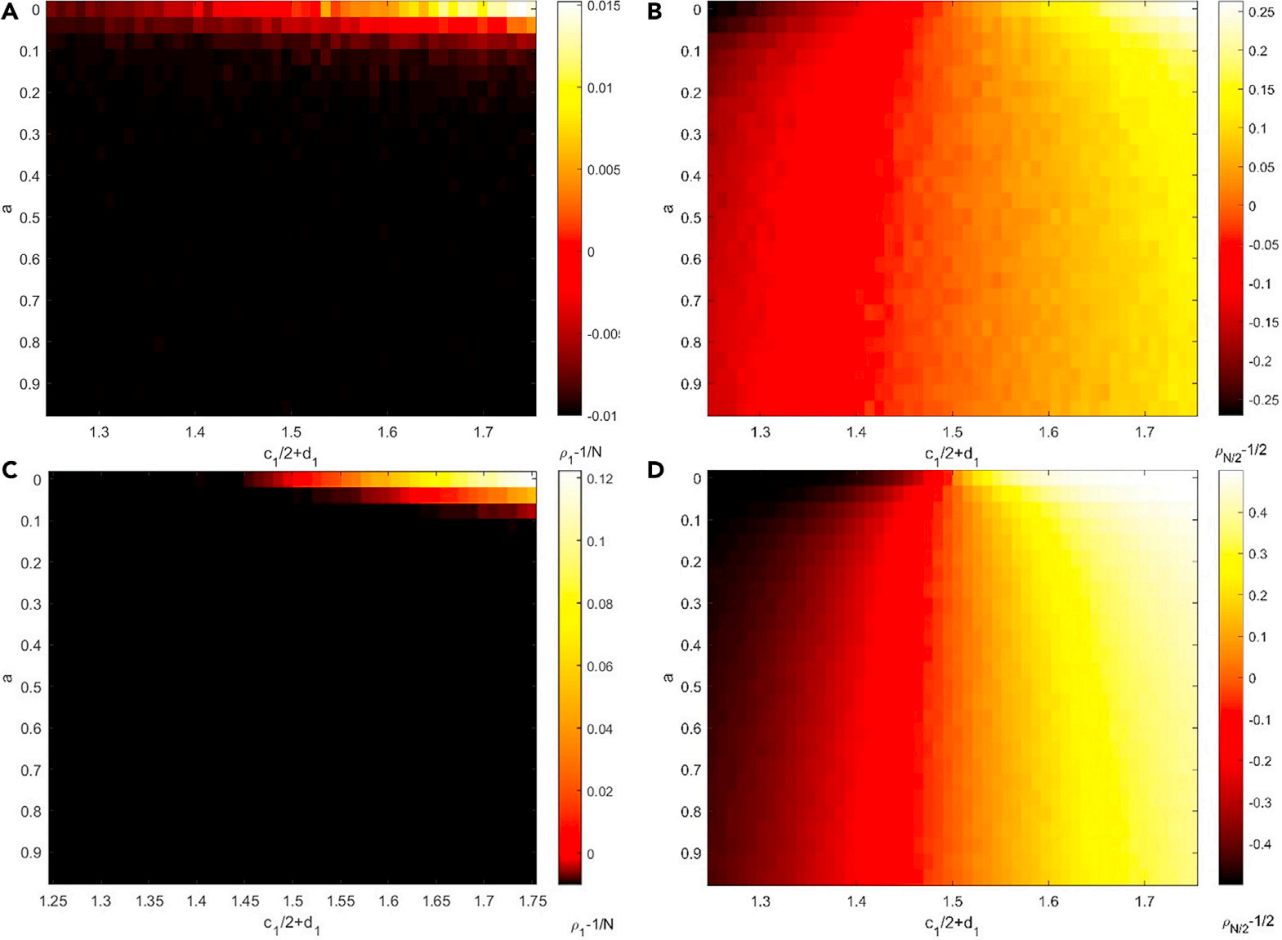
The degree of natural selection preference in the situation with positive feedback between environment and species (A–D) Positive feedback makes it difficult for species 1 to invade species 2, regardless of whether species 1 is initially dominating, neutral, or inferior in the invasion scenario no matter the selection intensity is (A) 0.1 or (C) 0.9. In a competing scenario, positive feedback could weaken the initially dominant species while strengthening the initially inferior species no matter the selection intensity is (B) 0.1 or (D) 0.9. Increasing intensity of feedback can strengthen these effects of positive feedback. The selection intensity doesn’t change these trends. The entire population size is 100. Each simulation is conducted a total of 10000 times to compute the probabilities $\rho_{\frac{1}{2}}$ and $\rho_{\frac{1}{N}}$, and data are represented as mean. Other parameter: Δt=0.01 and $\frac{c_{2}}{2}+d_{2}=1.5$.

Positive feedback increases the importance of individual numbers, therefore having a high individual number is advantageous. In the invasion scenario, the fixation probability $\rho_{1}$ declines fast as the intensity of feedback increases, regardless of whether species 1 is initially dominating, neutral, or inferior (Figures 2A and 2C). Positive feedback could be enhanced further by high selection intensity. In other words, positive feedback makes the invasion more difficult than in the absence of feedback, or perhaps impossible. The possibility of the dominant species taking over the population is very low, therefore the initially dominating species that would be favored by natural selection in the absence of feedback are likewise not favored anymore. This occurs because positive feedback permits the species with initial dominance to modify the environment more quickly, making it more appropriate for this species. The improved environment causes the species to be more fit and has a greater chance of resisting invasive success. To be more explicit, we examine the first two-time steps of this process for $\frac{c_{1}}{2}+d_{1}=1.75$. If species 1 is chosen to generate offspring and species 2 is chosen to have a deceased individual in the first step, individuals in species 2 will have a better environment than individuals in species 1 in the following step, which should be the same if no feedback is provided. When compared to the situation without feedback, positive feedback reduces the possibility of species 1 taking over the population. Furthermore, the occurrence of other events in the first step has no impact on the probability of species 1 taking over the population. As a result, positive feedback reduced the expect fixation probability of species 1.

**Figure 3:**
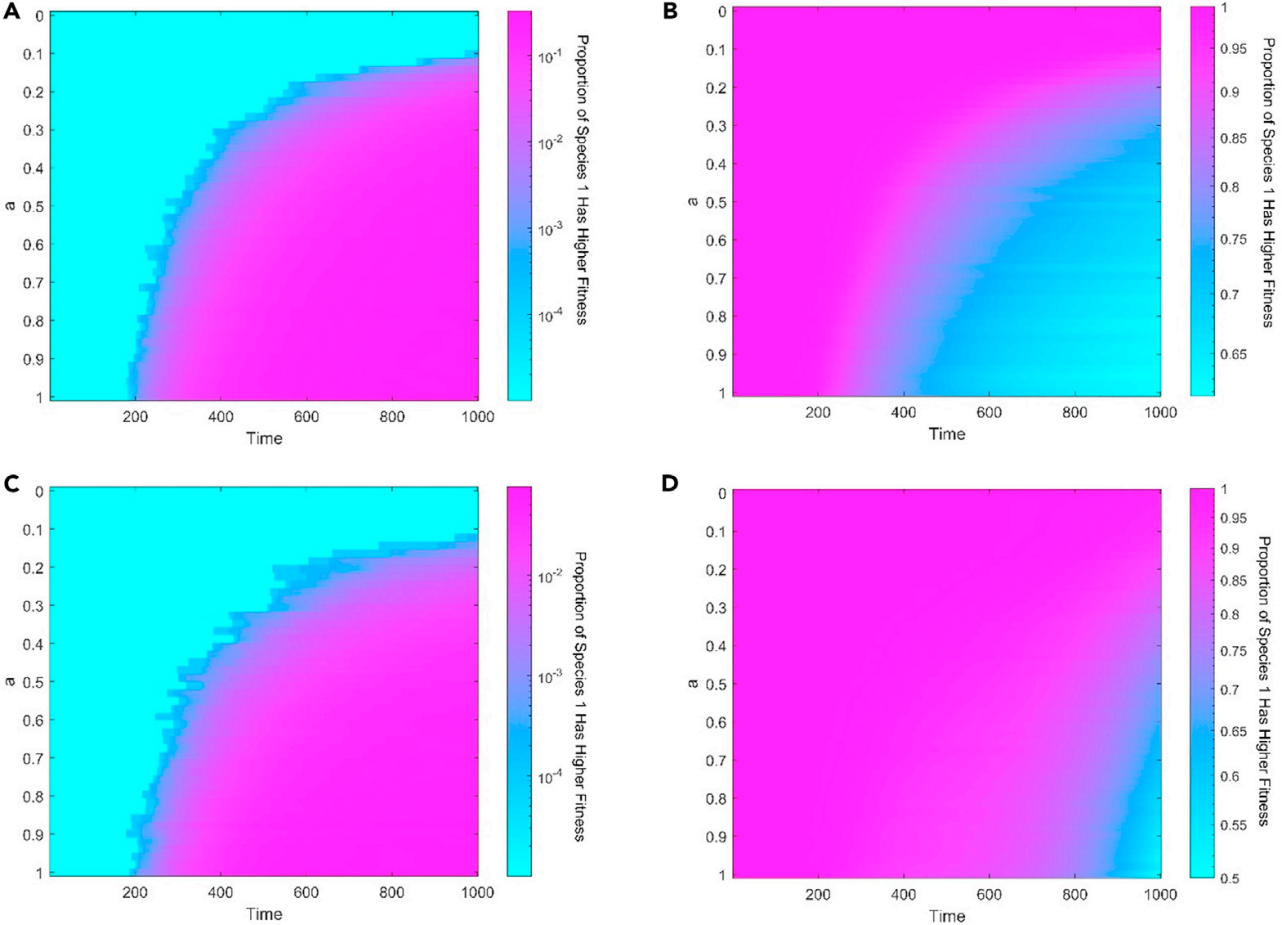
The effects of positive feedback on fitness at the early stage for competition scenario (A–D) Positive feedback allows initially inferior species 1 with $\frac{c_{1}}{2}+d_{1}=1.25$ to gain higher fitness than initial dominate species 2 no matter the selection intensity is (A) 0.1 or (C) 0.9. Similarly, positive feedback makes initial dominant species 1 with $\frac{c_{1}}{2}+d_{1}=1.75$ to lose its advantage in the beginning no matter the selection intensity is (B) 0.1 or (D) 0.9. The effect of positive feedback increases with the increasing intensity of feedback. High selection intensity doesn’t change these trends but weaken the effect of positive feedback. The entire population size is 100. Each simulation is conducted a total of 10000 times, and data are represented as mean. Other parameter: Δt=0.01 and $\frac{c_{2}}{2}+d_{2}=1.5$.

With positive feedback, the possibility of taking over the whole population decreases for the initially dominant species but increases for the initially inferior species with increasing intensity of feedback in the competing scenario (Figure 2B and 2D). In other words, positive feedback diminishes the dominant species’ dominance, making the inferior species more likely to take over the whole population than in the absence of feedback. Positive feedback, however, does not render species favored by natural selection unfavorable and vice versa. Because of demographic stochasticity, the originally inferior species may have more individuals, allowing it to have more resources and hence better fitness. More precisely, positive feedback makes species 1 have chance to have a greater fitness than species 2 in the early stages if species 1 is initially inferior (Figures 3A and 3C). Positive feedback makes early stochastic evolutionary paths so significant that they can affect the property of the evolution process. In other words, positive feedback makes stochasticity in the early stages of evolution more important. Similarly, positive feedback may causes species 1 to lose its advantage if it is the initial dominant species (Figure 3B and 3D). High selection intensity may reduce the probability of initial inferior species having more individuals than initial inferior species caused by demographic stochasticity.

### The situation with negative feedback

**Figure 4:**
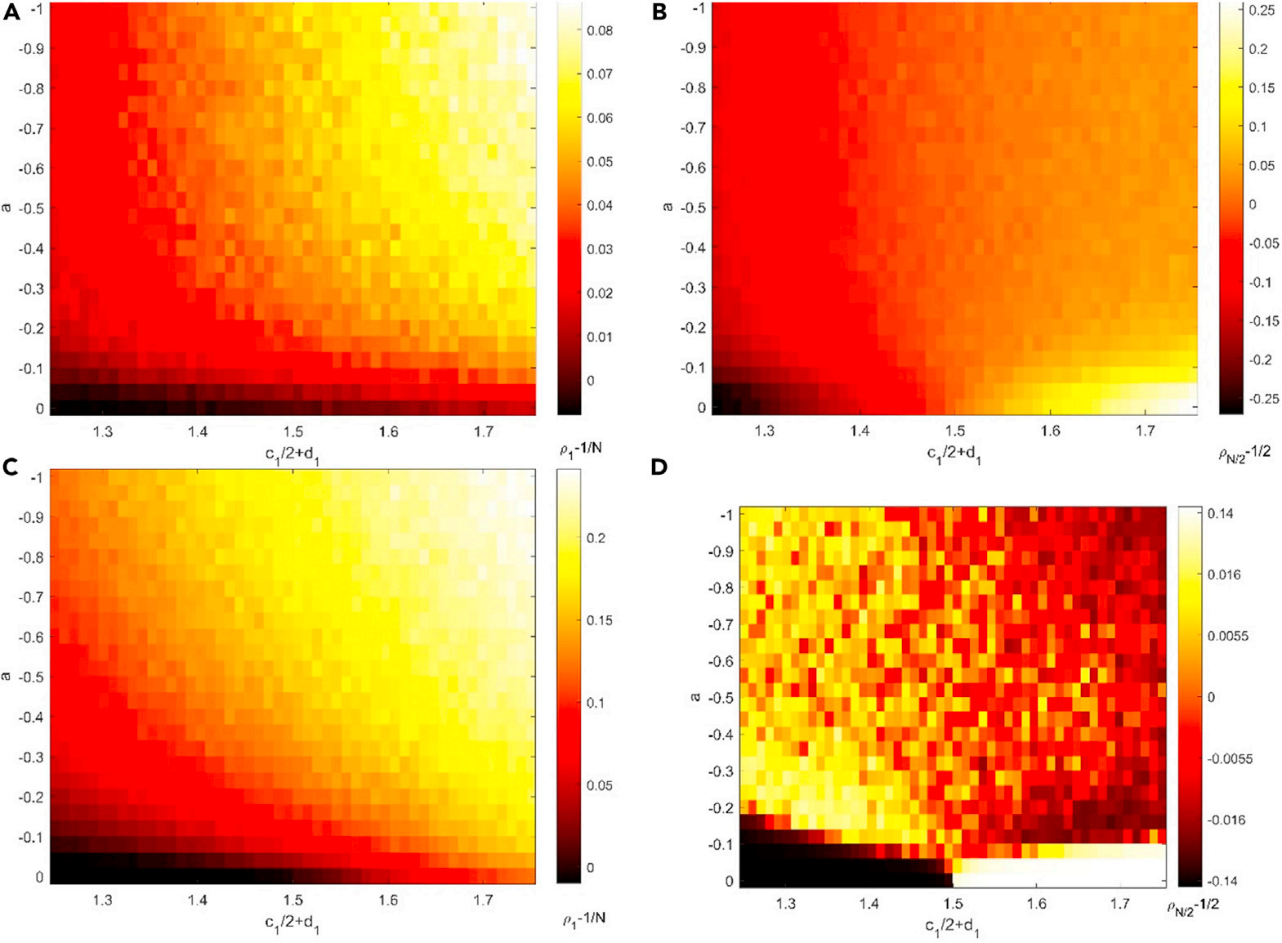
The degree of natural selection preference in the situation with negative feedback between environment and species (A–D) Negative feedback makes it easy for species 1 to invade species 2, regardless of whether species 1 is initially dominating, neutral, or inferior in the invasion scenario no matter the selection intensity is (A) 0.1 or (C) 0.9. Increasing intensity of feedback can strengthen these effects of negative feedback. The selection intensity doesn’t change these trends. In a competing scenario, Negative feedback could weaken the initially dominant species while strengthening the initially inferior species when the selection intensity is (B) 0.1 and even allow natural selection to favor initial inferior species when the selection intensity is (D) 0.9. The entire population size is 100. Each simulation is conducted a total of 10000 times to compute the probabilities $\rho_{\frac{1}{2}}$ , $\rho_{\frac{1}{N}}$ , and data are represented as mean. Proportion of species 1 has higher fitness than species 2 in first 500 time steps. Other parameter: Δt=0.001 and $\frac{c_{2}}{2}+d_{2}=1.5$.

Individual numbers become disadvantageous as a result of negative feedback. In the invasion scenario, negative feedback has the opposite effect on the outcomes as positive feedback. The fixation probability $\rho_{1}$ increases with increasing feedback intensity, regardless of whether species 1 is initially dominant, neutral, or inferior (Figures 4A and 4C). High selection intensity could further strengthen the effect of negative feedback. In particular, the fixation probability of initially inferior species may be more than 1/N, which is the fixation probability of neutral species in the absence of feedback. The rationale for this is that species with more starting individuals will consume resources more quickly, lowering the fitness of this species. The only individual from species 1 then obtains an advantage.

**Figure 5:**
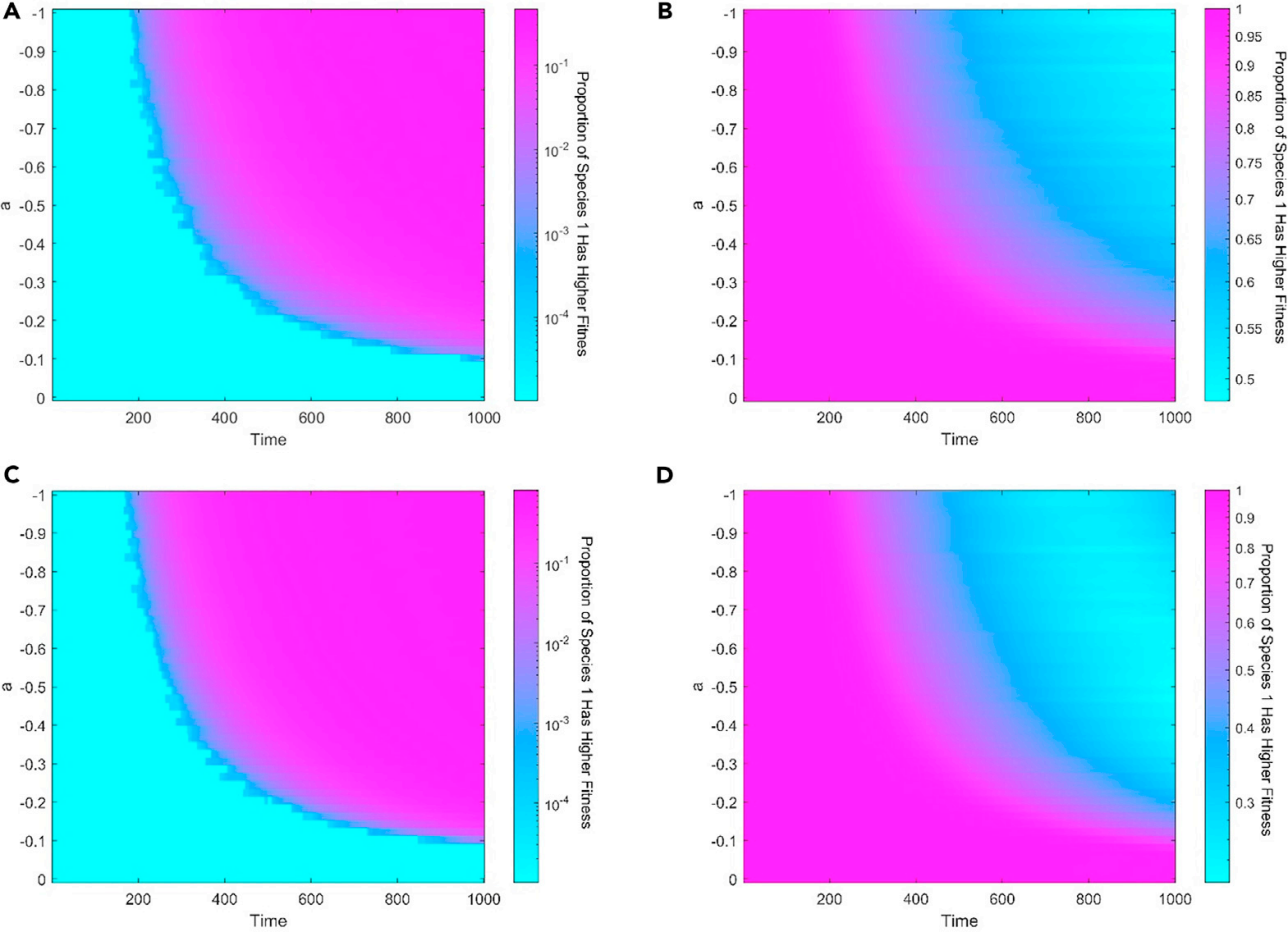
The effects of negative feedback on fitness at the early stage for competition scenario (A–D) Negative feedback allows initially inferior species 1 with $\frac{c_{1}}{2}+d_{1}=1.25$ to gain higher fitness than initial dominate species 2 no matter the selection intensity is (A) 0.1 or (C) 0.9. Similarly, negative feedback makes initial dominant species 1 with $\frac{c_{1}}{2}+d_{1}=1.75$ to lose its advantage in the beginning no matter the selection intensity is (B) 0.1 or (D) 0.9. The effect of negative feedback increases with the increasing intensity of feedback. High selection intensity doesn’t change these trends but weaken the effect of negative feedback. The entire population size is 100. Each simulation is conducted a total of 10000 times, and data are represented as mean. Other parameter: Δt=0.01 and $\frac{c_{2}}{2}+d_{2}=1.5$.

In a competition scenario, negative feedback serves the same effect as positive feedback when the selection intensity is weak. With increasing feedback intensity, the chance of taking over the whole population decreases for the initially dominant species but increases for the initially inferior species (Figure 4B). Similarly, negative feedback has no impact on whether the species is favored by natural selection. In other words, the possibility of an initially dominant species gaining control of the entire population will not be less than 1/2, and the probability of an initially inferior species gaining control of the entire population will not be more than 1/2. Negative feedback, like positive feedback, causes initially inferior species to have a high probability of fitness and initially dominating species to have a low fitness (Figure 5). Furthermore, with high selection intensity, the situation becomes more complicated. Natural selection favors initial inferior species (Figure 4D). The poor environment faced by species with more individuals reduces the birth rate of this species and thus prevents further increases in population size due to the increased influence of the environment on the birth rate of the species. This closes the fixation probability to 0.5. Because of the environment’s strong influence on the initially dominant species, the initially dominant species faces more resistance when the number of individuals is large, reducing the initially dominant species’ fixation probability. In other words, the initial inferior species with more disadvantages will instead have a higher fixation probability.

## Discussion

The evolutionary path often has little influence on the outcome in traditional natural selection theory, especially when there is just one peak on the static fitness landscape, and nature selection is regarded as a separate process that does not take historical accidents into consideration. The fitness landscape, on the other hand, is generally not static but varies, like a fitness seascape where selection changes on micro-evolutionary timescales.^30^ Since the effects of species on the environment, which in turn affects species fitness, are considered, study on feedback between species and environment is a specific instance of fluctuant fitness seascape.^31^ Here, we studied the importance of feedback in two crucial ecological scenarios. To begin, invasion scenarios depict the process by which a single individual from species 1 invades a community of individuals from species 2. Then, competition scenarios depict the process that occurs in areas of overlap in the distribution of two species that have an equal number of individuals at the start.

In the invasion scenarios, natural selection favors species 1 if its fitness is greater than that of species 2 when there is no feedback between environment and species. Positive feedback, on the one hand, makes invasion more difficult, so that even species with high fitness at the start will not be favored by natural selection. Because primordial species have an advantage in terms of individual quantity, the primordial species may be able to make the environment more conducive to itself. Negative feedback, on the other hand, makes large individual number a disadvantage and makes invasion more likely, so that even species with poor fitness may be favored by natural selection. Furthermore, the high intensity of feedback could strengthen the effect of positive feedback and negative feedback and selection intensity does not change these trends. These findings are consistent with experimental findings that nutrient availability can reinforce dominant species’ competitive ability and stabilize current vegetation if there is positive feedback between litter effect and nutrient availability, or it can enhance a potential competitor’s advantage and lead to vegetation change if there is a negative feedback between litter effect and nutrient availability.^32^^,^^33^^,^^34^

If there is no feedback in the competitive scenarios, species with higher fitness have a greater chance of taking over the whole population. However, both positive and negative feedback might benefit the initially inferior species, increasing its chances of taking over the entire population. In addition, the high intensity of feedback could further strengthen the effect of feedback. The explanation for this is because feedback allows originally inferior species to achieve high fitness. It emphasizes some of the early random events. In other words, in the presence of feedback, when the present state is dependent on the prior status, the evolutionary path is extremely important. This feature means that even initially inferior species have a chance to achieve high fitness without resorting to traditional genetic drift provided they are lucky enough to have a sufficient number of individuals at the beginning, even if they are of low fitness. As a result, the species that dominates the population does not always have the best fitness at the start. Another more interesting thing is negative feedback could make natural selection favor initial inferior species when the selection intensity is strong since the initial dominate species may face higher fitness disadvantage from individual number than the initial inferior species.

Natural selection research typically considers the effects of environment on species. In contrast, research on ecological niche construction has received increased attention and has placed more emphasis on the effects of species on the environment.^12^ When these two ideas are combined into a single framework, the role of feedback cannot be overlooked, and feedback-related research is on the rise.^19^^,^^22^^,^^35^ Our research looked at stochasticity in the context of positive or negative feedback. We believe this will be a fruitful direction.

### Limitations of the study

Our research is limited to the most basic scenario with two similar species and related environments. More complex scenarios should be considered in future research. A potential future question worth investigating is whether evolution is reproducible if the life’s tape is repeated, as proposed by Gould.^36^ The effects of stochasticity and feedback between environment and species may produce results that fall somewhere between complete randomness, which is consistent with Gould’s theory, and complete certainty, which is consistent with Darwin’s theory of natural selection. In other words, it promises to bring different perspectives on this issue together.

## STAR★Methods

### Key resources table

**Table:** 

REAGENT or RESOURCE	SOURCE	IDENTIFIER
**Deposited data**		

Code	This paper	https://github.com/crazynorthwest/iScience_2024_path_dependence.git

**Software and algorithms**		

MATLAB	R2023a, the Mathworks	https://matlab.mathworks.com/

### Resource availability

#### Lead contact

Further information should be directed to and will be fulfilled by the lead contact, Rui-Wu Wang (wangrw@nwpu.edu.cn).

#### Materials availability

This study did not generate new unique reagents.

#### Data and code availability

All data reported in this paper will be shared by the lead contact upon request.

The code used for simulation in this study is available at github: https://github.com/crazynorthwest/iScience_2024_path_dependence.git.

Any additional information required to reanalyze the data reported in this paper is available from the lead contact upon request.

### Method details

#### Without feedback

The detailed explanations of the model have been described in the main text. To reproduce the results in this paper, please use the code we have uploaded. The file ‘without_feedback.m’ is used to simulate the evolution of species without feedback with environment. By setting the code in line 12 in the file ‘without_feedback.m’ to

>population_size = 1;

and

>population_size = 50;

one can get the results for the invasion and competition scenarios, respectively (Figures 1A and 1B)

#### With positive feedback

The file ‘with_feedback_main.m’ is used to simulate the evolution of species with feedback with environment. The file ‘with_feedback_ early_stage.m’ is used to simulate the early stage in the evolution of species with feedback with environment. To obtain the simulation results for the positive feedback condition, it is necessary to set the 18th line of code in ‘with_feedback_main.m’ and ‘with_feedback_early_stage.m’ to

>parameter_a_collection = linspace(0,1,parameter_idx)';

Also in these two files, the code in the 17th line represents the Selection intensity in the simulation. The results for the invasion and competition scenario can be gotten by setting the 12th line of code in the file ‘with_feedback_main.m’ to

>population_size = 1;

and

>population_size = 50;

respectively. For the results in early stage of competition scenario, the code in 19th line and 47th line in the file ‘with_feedback_ early_stage.m’ need to be set to

>parameter_c(1) = 0.5;

and

>result_sum_env(idx3,) = mean(sign(sign(1.5∗result_record_env-1)+1));

to simulate the inferior species, or >parameter_c(1) = 0.5;

and

>result_sum_env (idx3,) = mean(sign(sign(2.5∗result_record_env-1)+1));

to simulate the initially dominating species.

#### With negative feedback

To obtain the simulation results for the positive feedback condition, it is necessary to set the 18th line of code in ‘with_feedback_main.m’ and ‘with_feedback_early_stage.m’ to.

>parameter_a_collection = linspace(-1,0,parameter_idx)';

Other settings are the same as in the case of positive feedback.

### Quantification and statistical analysis

All simulations were done with MATLAB R2023a. All simulations were repeated 10000 times and the mean value of these results were shown in figures. No other statistical methods were used to determine whether the data met assumptions of the statistical approach.
